# A retrospective study of the clinical phenotype and predictors of survival in non-Caucasian Hispanic patients with amyotrophic lateral sclerosis

**DOI:** 10.1186/s12883-019-1459-3

**Published:** 2019-10-29

**Authors:** Claudia Marisol Sánchez-Martínez, José Alberto Choreño-Parra, Lilia Nuñez-Orozco, Noel Placencia-Álvarez, Laura Marcela Alvis-Castaño, Parménides Guadarrama-Ortiz

**Affiliations:** 1 Department of Neurology, Centro Médico Nacional 20 de Noviembre, ISSSTE, Félix Cuevas, 540, Col del Valle Sur, 03100 Mexico City, Mexico; 2 Department of Research, Centro Especializado en Neurocirugía y Neurociencias México (CENNM), Mexico City, Mexico; 3 Escuela Nacional de Ciencias Biológicas, Instituto Politécnico Nacional, Mexico City, Mexico; 4 Department of Neurosurgery, Centro Especializado en Neurocirugía y Neurociencias México (CENNM), Tlaxcala & Manzanillo, Roma Sur, 06760 Mexico City, Mexico

**Keywords:** Amyotrophic lateral sclerosis, Motor neuron disease, Spinal-onset ALS, Bulbar-onset ALS, Prognostic factors

## Abstract

**Background:**

Little is known about the clinical phenotype of amyotrophic lateral sclerosis (ALS) in non-Caucasian populations. Here, we aimed to describe the clinical characteristics, prognostic factors and survival of Mexican patients with ALS.

**Methods:**

We conducted a retrospective study by reviewing the medical records of patients with ALS that attended and were regularly followed at a third level hospital in Mexico City from 2000 to 2015. We calculated absolute and relative frequencies of the clinical characteristics from all the participants. We also estimated correlation coefficients between clinical features and overall survival. Additionally, survival rates were compared for all participants grouped according to different clinical features using the Kaplan-Meier method and the log-rank test.

**Results:**

We enrolled 45 ALS patients, 53.33% had spinal-onset ALS and 46.66% presented bulbar ALS. The male/female ratio was 0.8. The mean age at onset of symptoms was 58.11 years. Mean survival time from onset was 64.73 ± 34.83 months. Cumulative survival rate after 5 years of disease onset was 44.44%. Age at onset and age at diagnosis inversely correlated with overall survival time. Also, we found that bulbar-onset, short diagnostic delay, percutaneous endoscopic gastrostomy, mechanical ventilation, and lower total cholesterol serum levels were associated with short survival.

**Conclusions:**

The clinical characteristics of Mexican ALS patients differ from the disease phenotype observed in Caucasians. Nonetheless, the predictive value of certain well-recognized prognostic factors remains consistent in our population. The current study provides relevant information for a better understanding of prognostic factors in ALS patients from Mexico and other Latin American countries.

## Background

Amyotrophic lateral sclerosis (ALS) is a fatal neurodegenerative disease characterized by the selective loss of motor neurons along different regions of the central nervous system, including the brain and the anterior horns of the spinal cord. This disorder manifests in the form of progressive painless weakness of several muscle groups including those involved in respiratory movements, which invariably leads to respiratory failure and death [1]. In addition, an increasing number of studies have recently reported sensory and autonomic symptoms in ALS patients, supporting an involvement of the peripheral nervous system in the pathogenic mechanisms of this disease [2]. Hence, the clinical spectrum of ALS is heterogeneous and encompasses several forms of the disease that differ in their neurological phenotype.

Adding complexity to the clinical landscape of ALS, it is becoming widely recognized that the frequency and clinical characteristics of ALS are influenced by the presence of genetic abnormalities [3], and also vary according to ethnicity [4], showing stable incidence and clinical features among Caucasians [4–6]. This suggests that the frequency of clinical and demographic characteristics with prognostic significance may also vary according to the race of ALS patients. However, little is known about the incidence and clinical phenotype of ALS among other groups. Thus, more studies evaluating clinical characteristics and prognostic factors associated with survival in non-white Caucasian individuals with ALS are needed. For such purpose, we performed a retrospective study of the clinical features and survival of Hispanic ALS patients from Mexico City. Our results confirmed the well-known prognostic value of some clinical variables previously described in Caucasian series. We also showed certain particularities in the clinical phenotype of our population. Hence, our study may provide relevant information for the specific approach to ALS patients from Mexico and other Latin American countries.

## Methods

### Participants

We reviewed the medical records of all the individuals with ALS that were admitted and regularly followed at the Centro Médico Nacional “20 de Noviembre” of the Instituto de Seguridad y Servicios Sociales de los Trabajadores del Estado (ISSSTE) in Mexico City, from January of 2000 to December of 2015. We enrolled patients with clinical findings of probable or definitive ALS according to the “El Escorial” diagnostic criteria [7], as well as those with electrophysiological signs of motor neuron involvement compatible with probable or definitive ALS as proposed by the Awaji-shima consensus recommendations [8]. Patients with electrophysiological, neuroimaging or pathological evidence of other disease processes causing the motor neuron degeneration were ineligible. Clinical and demographic data of all participants were retrieved from our medical record database. The obtained information included age at onset, age at diagnosis, gender, weight at onset, body weight loss after diagnosis, anatomical region of disease onset, relevant comorbidities, family history of ALS, onset to diagnosis interval (ODI), diagnosis to death interval (DDI), major medical interventions such as percutaneous endoscopic gastrostomy (PEG) secondary to dysphagia, need for mechanical ventilation (MV) secondary to respiratory failure, onset to gastrotomy interval (OGI), gastrostomy to death interval (GDI), onset to mechanical ventilation interval (OMVI), mechanical ventilation to death interval (MVDI), time of survival from disease onset, cause of death, and total cholesterol serum levels at diagnosis. The survival and other clinical data from patients that remained alive were censored at the last known follow-up. No genetic studies were performed in our patients looking for the presence of genetic abnormalities known to be involved in ALS.

### Statistical analysis

For descriptive statistics, we calculated absolute and relative frequencies of the clinical characteristics from all the participants, overall and grouped according to the anatomical region of disease onset: spinal-onset or bulbar-onset. Continuous variables were reported as medians, means, standard deviation (SD) or 95% confidential intervals (95% CI). Categorical variables were reported as percentages. Differences in categorical variables between spinal-onset and bulbar-onset participants were assessed by Fisher exact or Chi square test, whereas comparisons of continuous variables were performed using the Student-T test or Mann-Whitney U test, as appropriate. To estimate the impact of different clinical variables on the overall patients’ survival, we first estimated correlations between continuous clinical variables and overall survival time using the Pearson or Spearman correlation coefficient. Additionally, survival rates were compared for all participants, as well as for separated spinal- and bulbar-onset groups, according to the different clinical features evaluated. For this purpose, we used the Kaplan-Meier method and the log-rank test to compare two survival curves, or the log-rank test for trend for comparisons between three survival curves. Finally, to further estimate the magnitude of the association between clinical features and survival, we divided participants in two groups depending on whether their survival was found below or above the overall mean. Then, we calculated the prognostic value of each clinical factor expressed in terms of Odds ratio (OR) for poor survival using a bivariate logistic regression analysis. We also compared continuous clinical variables between subjects with short and long survival by Student-T test or Mann-Whitney U test. All analyses were performed using the software package GraphPad Prism Version 8.0 (San Diego, CA, USA). A *p* value <0.05 was considered statistically significant.

### Ethical statements

The current study was reviewed and approved by the Ethics committee of the Centro Médico Nacional “20 de Noviembre”, Instituto de Seguridad y Servicios Sociales de los Trabajadores del Estado (ISSSTE) in Mexico City.

## Results

### Participants characteristics

We enrolled 45 ALS patients, 20 males and 25 females; the male/female ratio was 0.8. From these, 53.33% had spinal-onset whereas 46.66% presented bulbar ALS. Their mean age at onset of symptoms was 58.11 years. Thirteen patients had other relevant comorbidities, among which systemic arterial hypertension (SAH) and ischemic cardiopathy were the most frequent. Only 2 participants had family history of ALS. All the patients had at least one first-degree relative with indigenous ethnic background. Any participant received treatment with riluzole. Clinical and demographic characteristics of enrolled individuals are summarized in Table 1.

**Table 1:** Clinical characteristics of the study participants

Clinical characteristics	Total participants	Spinal onset	Bulbar onset	*p* value
		(*n* = 24)	(*n* = 21)	
	(*n* = 45)			
Age at onset (years), mean (range)	58.11 (38–82)	55.96 (42–82)	60.57 (38–81)	0.1566
Age at diagnosis (years), mean (range)	58.89 (40–83)	58.17 (43–83)	61.86 (40–81)	0.2432
Gender				
Males, *n* (%)	20 (44.44)	12 (50)	8 (38.09)	0.4227
Females, *n* (%)	25 (55.55)	12 (50)	13 (61.90)	
Weight at diagnosis (Kg), mean (SD)	50.11 (12.06)	48.61 (12.18)	51.07 (12.34)	0.6536
Family history of ALS, *n* (%)	2 (4.44)	1 (4.16)	1 (4.76)	0.4227
Comorbidities, *n* (%)	13 (28.88)	6 (25)	7 (33.33)	0.9230
SAH, *n* (%)	8 (17.77)	4 (16.66)	4 (19.04)	0.5384
Ischemic cardiopathy, *n* (%)	3 (6.66)	1 (4.16)	2 (9.52)	0.8349
Hypothyroidism, *n* (%)	2 (4.44)	1 (4.16)	0 (0)	0.4723
Diabetes, *n* (%)	1 (2.22)	1 (4.16)	0 (0)	0.4723
Cancer, *n* (%)	1 (2.22)	0 (0)	1 (4.76)	0.2796
Rheumatoid arthritis, *n* (%)	1 (2.22)	0 (0)	1 (4.76)	0.2796
Gout, *n* (%)	1 (2.22)	1 (4.16)	0 (0)	0.4723
Weight loss (Kg), mean (SD)	11.79 (6.12)	10.83 (4.44)	11.76 (8.29)	0.8334
Survival time from onset (mo), mean (SD)	64.73 (34.83)	73.96 (35.60)	54.19 (31.51)	0.0565
ODI (mo), mean (SD)	22.22 (16.51)	27.50 (19.96)	16.19 (8.34)	0.0483
DDI (mo), mean (SD)	42.51 (28.44)	46.46 (30.34)	38 (26.09)	0.3252
PEG, *n* (%)	31 (68.88)	17 (70.83)	14 (66.66)	0.5384
OGI (mo), mean (SD)	18.61 (18.03)	20.47 (21.65)	16.36 (12.79)	0.9291
GDI (mo), mean (SD)	44.90 (28.50)	48.88 (31.74)	40.07 (24.27)	0.4009
MV, *n* (%)	32 (71.11)	18 (75)	14 (66.66)	0.4723
OMVI (mo), mean (SD)	26.84 (25.12)	34.94 (30.40)	16.43 (9.37)	0.0266
MVDI (mo), mean (SD)	29.69 (24.41)	28.33 (21.95)	31.43 (28.03)	0.8880
Cause of death				
Respiratory failure, *n* (%)	28 (62.22)	17 (70.83)	11 (52.38)	0.2028
Pneumonia, *n* (%)	11 (24.44)	4 (16.66)	7 (33.33)	0.1943
Sudden cardiac arrest, *n* (%)	3 (6.66)	1 (4.16)	2 (9.52)	0.4723
Myocardial infarction, *n* (%)	2 (4.44)	1 (4.16)	1 (4.76)	0.4227
Abdominal sepsis, *n* (%)	1 (2.22)	1 (4.16)	0 (0)	0.4723
Total cholesterol levels at onset (mmol/L), mean (SD)^a^	4.63 (1.11)	4.66 (1.29)	4.58 (0.86)	0.8588

^a^ These data were available only for 26 participants. Values of *p* were estimated with the Fisher exact Xi^2^ test and Student-T test or Mann-Whitney U test. *DDI* diagnosis to death interval, *Kg* kilograms, *mo* months, *mmol/L* millimoles per liter, *MV* mechanical ventilation, *ODI* onset to diagnosis interval, *OGI* onset to gastrostomy interval, *OMVI* onset to mechanical ventilation interval, *PEG* percutaneous endoscopic gastrostomy, *SD* standard deviation, *SAH* systemic arterial hypertension

### Survival of patients with ALS

Mean survival time since onset of disease in our ALS patients was 64.73 ± 34.83 months, whereas mean survival time after diagnosis was 42.51 ± 28.44 months. Spinal-onset patients had a longer survival time compared to bulbar-onset ALS patients (Table 1). Respiratory failure was the most common cause of death observed in our population followed by pneumonia. Indeed, 71.11% of enrolled individuals required MV during the period of the study, whereas 68.88% were subjected to PEG. We did not find differences in the number of patients requiring such major medical interventions between spinal- and bulbar-onset groups, but the OMVI was significatively shorter in individuals with bulbar ALS (Table 1). On the other hand, we estimated cumulative survival rates of all ALS patients and grouped according to their site of disease onset. At 1 year after disease onset the overall survival rate was 95.55%, however, at this time point, deaths accounted only for bulbar-onset ALS cases, as 100% of spinal-onset patients remained alive (Table 2). Cumulative survival at 5 years was 44.44% for all ALS patients, 54.16% for spinal-onset cases, and 33.33% for bulbar-onset patients.

**Table 2:** Cumulative survival rates in patients with ALS

Years	Survival (%, 95 CI)		
	Total participants	Spinal onset	Bulbar onset
1 year	95.55 (3.31–12.17)	100	90.47 (7.05–23.47)
2 years	88.88 (6.32–13.54)	100	76.19 (13.13–24.25)
3 years	71.11 (10.97–15.60)	75.00 (12.90–22.38)	66.66 (15.83–24.13)
4 years	53.33 (13.23–15.46)	58.33 (16.65–21.88)	47.61 (19.05–21.90)
5 years	44.44 (13.71–14.72)	54.16 (17.26–21.45)	33.33 (19.74–18.45)

Survival rates and their 95% CI were estimated using the log rank test for trend

### Impact of clinical features on survival of ALS patients

**Fig. 1:**
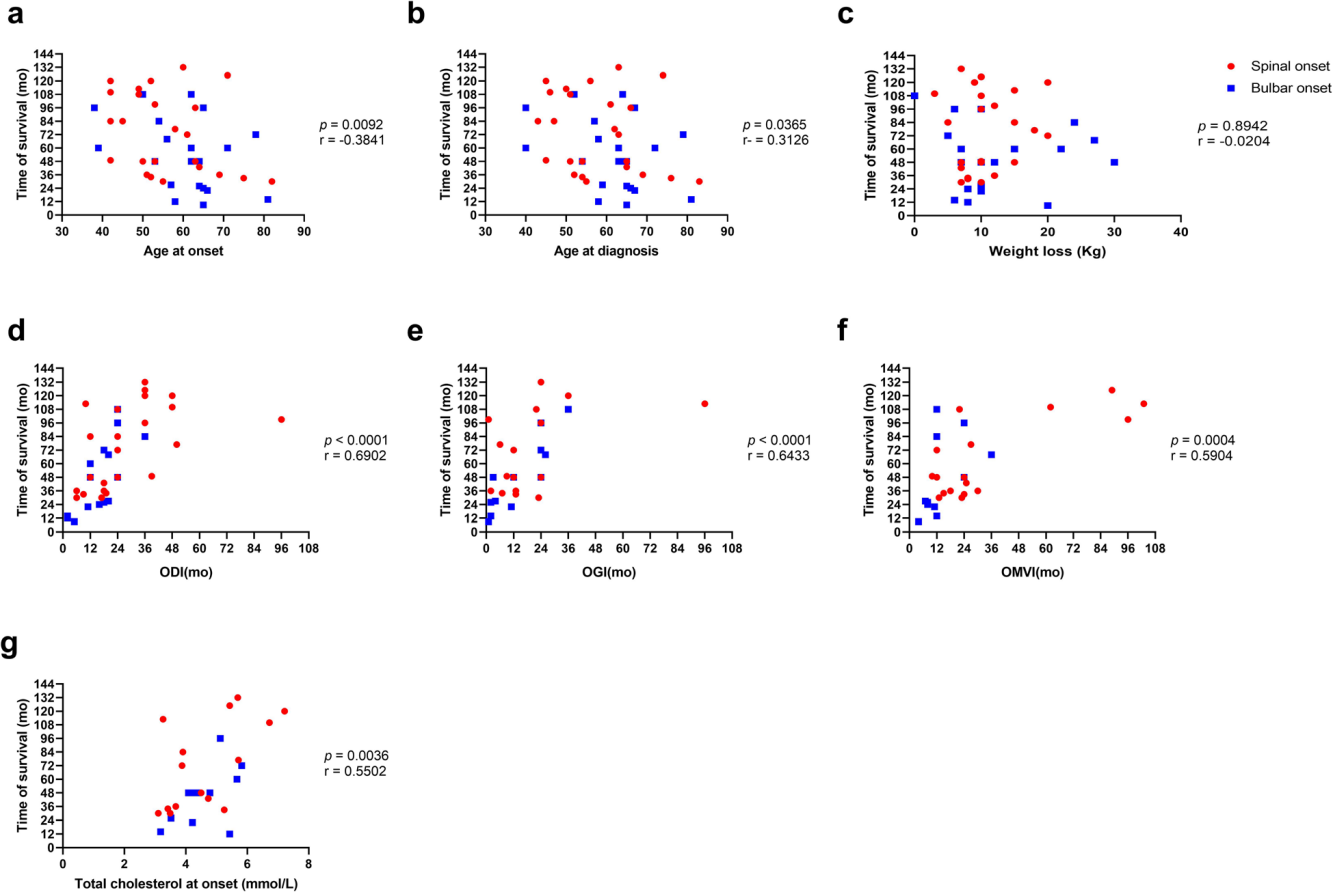
Correlations between continuous clinical variables and survival of patients with ALS. Linear correlations between different clinical variables and survival are shown. Age at onset (**a**); age at diagnosis (**b**); weight loss (**c**); ODI (**d**); OGI (**e**); OMVI (**f**), total cholesterol at onset (**g**). Values of *p* and r were estimated using the Pearson or Spearman correlation coefficients according to the distribution of data. Kg, kilograms; mo, months; ODI, onset to diagnosis interval; OGI, onset to gastrostomy interval; OMVI, onset to mechanical ventilation interval

**Fig. 2:**
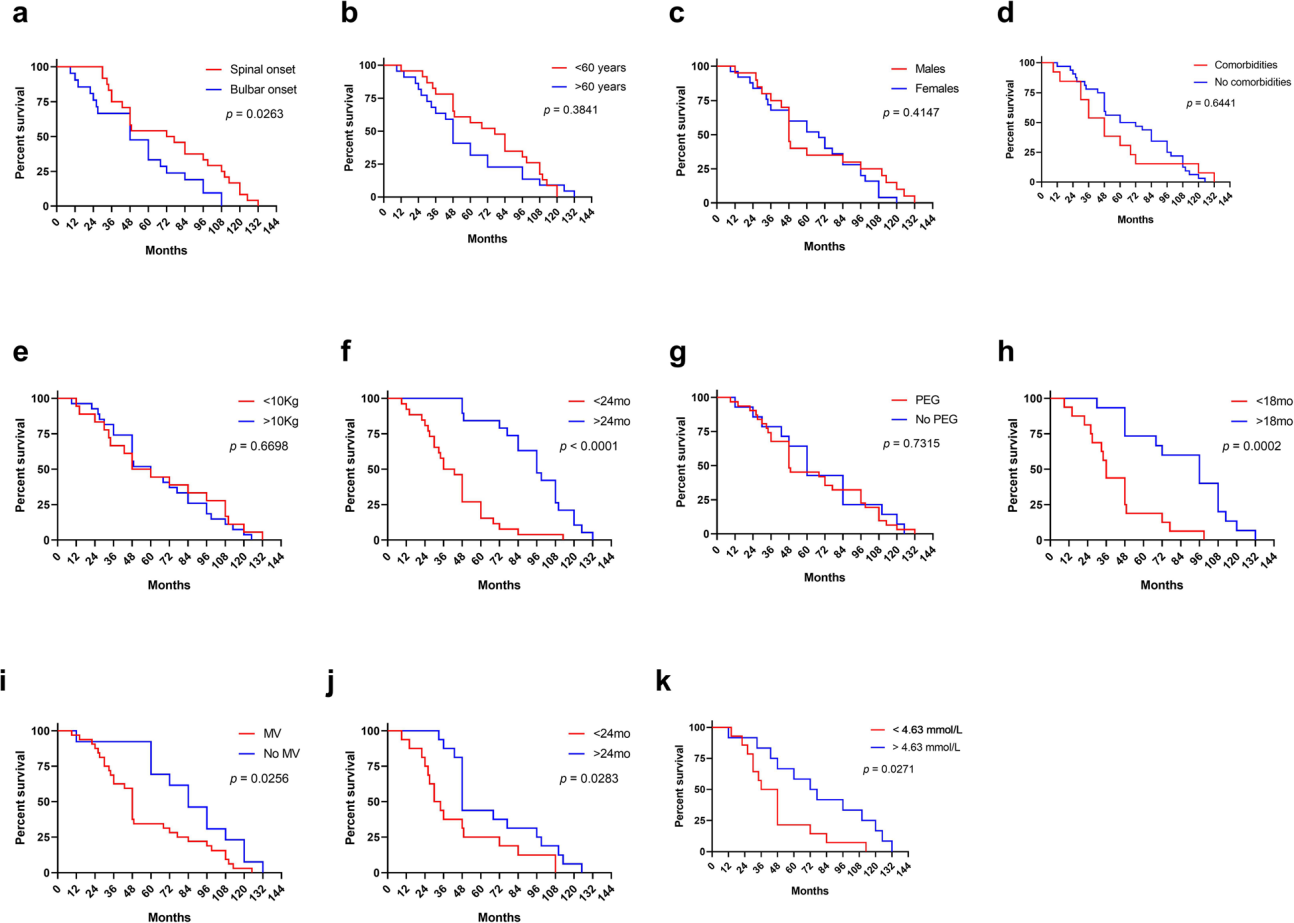
Clinical variables affecting overall survival of patients with ALS. Survival curves of ALS patients grouped according to the site of disease onset (**a**), age group (**b**), gender (**c**), presence of comorbidities (**d**), weight loss (**e**), ODI (**f**), PEG (**g**), OGI (**h**), MV (**i**), OMVI (**j**), and total cholesterol at onset (**k**). Differences in survival between groups were estimated with the log-rank test. Kg, kilograms; mo, months; MV, mechanical ventilation; ODI, onset to diagnosis interval; OGI, onset to gastrostomy interval; OMVI, onset to mechanical ventilation interval; PEG, percutaneous endoscopic gastrostomy

Using a linear correlation analysis, we found that age at onset and age at diagnosis inversely correlated with overall survival time, whereas ODI, OGI, and OMVI showed a positive correlation with such outcome (Fig. 1). We also compared survival curves of ALS patients categorized according to different clinical variables. This analysis revealed that the site of disease onset significatively affected the survival, as spinal-onset ALS patients had a longer survival than individuals with the bulbar variant (Fig. 2a**,** Table 3). Interestingly, we also found that survival was significatively longer for patients with delayed diagnosis compared to those with an ODI < 24 months (*p* < 0.0001; Fig. 2f**,** Table 3). This could be related to a trend for older ALS patients to have a shorter ODI (Additional file 1: Figure S1) and a shorter survival (Fig. 1, Table 3).

**Table 3:** Clinical characteristics affecting overall survival of patients with ALS

Clinical characteristic	Total participants (*n* = 45)	Survival time from onset (mo), mean (95% CI)	Log rank *p* value	Spinal onset (*n* = 24)	Survival time from onset (mo), mean (95% CI)	Log rank *p* value	Bulbar onset (*n* = 21)	Survival time from onset (mo), mean (95% CI)	Log rank *p* value
Age at onset, *n* (%)									
< 60 years, *n* (%)	23 (51.11)	72.30 (57.84–86.77)	0.3841	15 (62.50)	77.33 (58.71–95.95)	0.8671	8 (38.09)	62.88 (35.13–90.62)	0.3895
> 60 years, *n* (%)	22 (48.88)	56.82 (41.20–72.44)		9 (37.50)	68.33 (37.51–99.15)		13 (61.90)	48.85 (30.41–67.28)	
Gender									
Males, *n* (%)	20 (44.44)	65.15 (47.57–82.73)	0.4147	12 (50)	78.17 (53.11–103.20)	0.1925	8 (38.09)	45.63 (24.14–67.11)	0.2401
Females, *n* (%)	25 (55.55)	64.40 (50.67–78.13)		12 (50)	69.75 (49.10–90.40)		13 (61.90)	59.46 (38.61–80.31)	
Comorbidities									
Yes, *n* (%)	13 (28.88)	54.08 (31.67–76.48)	0.6441	6 (25)	64.00 (13.37–	0.7702	7 (33.33)	45.57 (22.43–68.71)	0.2386
No, *n* (%)	32 (71.11)	69.06 (56.98–81.14)		18 (75)	114.6) 77.28 (61.69–92.87)		14 (66.66)	58.50 (38.68–78.32)	
Weight loss									
<10 kg, *n* (%)	18 (40)	65.33 (45.89–84.78)	0.6698	9 (37.5)	70.44 (38.73–102.20)	0.8376	9 (42.85)	60.22 (30.57–89.88)	0.2239
>10 kg, *n* (%)	27 (60)	64.33 (51.50–77.17)		15 (62.5)	76.07 (57.73–94.41)		12 (57.14)	49.67 (33.21–66.12)	
ODI (mo)									
<24 mo, *n* (%)	26 (57.77)	43.58 (34.03–53.12)	< 0.0001	11 (45.83)	48.64 (30.99–66.28)	0.0010	15 (71.42)	39.87 (27.87–51.86)	0.0007
>24 mo, *n* (%)	19 (42.22)	93.98 (81.31–106.10)		13 (54.16)	95.38 (78.63–112.1)		6 (28.57)	90.00 (66.44–113.60)	
PEG									
Yes, *n* (%)	31 (68.88)	63.52 (50.63–76.40)	0.7315	17 (79.83)	69.35 (51.33–87.38)	0.4801	14 (66.66)	56.43 (36.13–76.73)	0.3952
No, *n* (%)	14 (31.11)	67.43 (47.04–87.82)		7 (29.16)	85.14 (50.87–119.40)		7 (33.33)	49.71 (27.13–72.30)	
OGI (mo)	(*n* = 31)			(*n* = 17)			(*n* = 14)		
<18 mo, *n* (%)	16 (51.61)	42.38 (29.69–55.06)	0.0002	9 (52.94)	53.78 (35.69–71.86)	0.0286	7 (50)	27.71 (13.60–41.83)	0.0006
>18 mo, *n* (%)	15 (48.38)	86.07 (68.72–103.40)		8 (47.05)	86.88 (54.34–119.40)		7 (50)	85.14 (64.03–106.30)	
MV									
Yes, *n* (%)	32 (71.11)	56.53 (44.75–68.32)	0.0256	18 (75)	63.28 (46.74–79.81)	0.0273	14 (66.66)	47.86 (29.99–65.73)	0.2478
No, *n* (%)	13 (28.88)	84.92 (65.18–104.7)		6 (25)	106.0 (84.31–127.70)		7 (33.33)	66.86 (38.30–95.41)	
OMVI (mo)	(*n* = 32)			(*n* = 18)			(*n* = 14)		
<24 mo, *n* (%)	16 (50)	45.06 (28.34–61.79)	0.0283	8 (44.44)	50.88 (28.31–73.44)	0.0953	8 (57.14)	39.25 (9.04–69.46)	0.3413
>24 mo, *n* (%)	61 (50)	68.00 (51.64–84.36)		10 (55.55)	73.20 (47.65–98.75)		6 (42.85)	59.33 (38.70–79.97)	
Total cholesterol at onset	(*n* = 26)		0.0271	(*n* = 15)		0.0496	(*n* = 11)		0.0860
< 4.63 mmol/L, *n* (%)	14 (53.84)	46.64 (31.14–62.15)		8 (53.33)	55.88 (30.23–81.52)		6 (54.54)	34.44 (18.11–50.56)	
>4.63 mmol/L, *n* (%)	12 (46.15)	77.33 (52.28–102.40)		7 (46.66)	91.43 (53.86–129.0)		5 (45.45)	57.60 (19.03–96.17)	

*Kg* kilograms, *mo* months, *mmol/L* millimoles per liter, *MV* mechanical ventilation, *ODI* onset to diagnosis interval, *OGI* onset to gastrostomy interval, *OMVI* onset to me chanical ventilation interval, *PEG* percutaneous endoscopic gastrostomy, *95% CI* 95% confidence interval

PEG secondary to dysphagia per se did not affect the overall survival of ALS patients, but individuals with OGI > 18 months had a significant longer survival compared with those requiring an early intervention (Fig. 2g-h**,** Table 3). Meanwhile, as expected, MV and a short OMVI (< 24 months) significatively worsened survival of ALS patients, especially in those with spinal-onset ALS (Fig. 2i-j**,** Table 3). Previous studies have demonstrated a positive correlation between levels of blood lipids measured at the time of diagnosis and survival of patients with ALS [9, 10]. Therefore, here we also evaluated the predictive significance of total cholesterol levels at diagnosis in our study population. Although we were able to collect this value only from 26 participants, we observed that higher levels of cholesterol at the time of diagnosis positively correlated with longer survival (Fig. 1g). Moreover, those patients with cholesterol levels below the overall mean (< 4.63 mmol/L) had a shorter survival rate (Fig. 2k). In addition, we observed that the mean value of cholesterol levels in patients requiring MV were lower compared to patients without respiratory failure (4.33 mmol/L vs 5.54 mmol/L, *p* = 0.0107; unpaired T test, data not shown). Similarly, serum cholesterol levels tended to be lower in patients underwent to PEG compared to patients that did not require such intervention (4.34 mmol/L vs 5.17 mmol/L, *p* = 0.0664; unpaired T test, data not shown).

Finally, to have an approximation of the magnitude of the effect of clinical characteristics on ALS prognosis, we grouped patients into two categories depending on whether their survival was found below or above the overall mean (~ 5 years). Then, we compared the means of continuous clinical variables between both groups and calculated the OR for short survival conferred by each categorical variable. Using this approach, we confirmed that a short ODI was associated with poor prognosis, with an OR for short survival of 14.48 (Table 4). We also found statistically significant ORs for short survival conferred by an OGI < 18 moths (OR = 11.92), necessity of MV (OR = 22.91), and lower serum levels of total cholesterol at diagnosis (OR = 7.33).

**Table 4:** Clinical features of ALS patients with short and long survival

Characteristic	Short survival (*n* = 22)	Long survival (*n* = 23)	OR (95% CI)
Age at onset (years), mean (range)	61.50 (42–82)	54.87 (38–78)	NA
Age group			0.91 (0.30–2.79)
< 60 years, *n* (%)	11 (50)	12 (52.17)	
> 60 years, *n* (%)	11 (50)	11 (47.82)	
Gender			2.25 (0.63–7.45)
Males, *n* (%)	12 (54.54)	8 (34.78)	
Females, *n* (%)	10 (45.45)	15 (65.21)	
Weight at diagnosis (Kg), mean (SD)	52.14 (11.61)	49.22 (12.52)	NA
Weight loss (Kg), mean (SD)	10.77 (5.30)	12.86 (6.83)	1.07 (0.35–3.32)
<10 kg, *n* (%)	9 (40.90)	9 (39.13)	
>10 kg, *n* (%)	13 (59.09)	14 (60.86)	
Comorbidities			0.48 (0.13–1.80)
Yes, *n* (%)	8 (36.36)	5 (21.73)	
No, *n* (%)	14 (63.63)	18 (78.26)	
ODI (mo), mean (SD)	14.27 (8.44)	*** 29.83 (18.80)	14.48 (3.38–53.99)
<24 mo, *n* (%)	19 (86.36)	7 (30.43)	
>24 mo, *n* (%)	3 (13.63)	16 (69.56)	
PEG, *n* (%)	17 (77.27)	14 (60.86)	2.18 (0.62–7.65)
OGI (mo), mean (SD)	10.94 (8.43)	**27.93 (22.20)	11.92 (2.27–50.84)
<18 mo, *n* (%)	13 (76.47)	3 (21.42)	
>18 mo, *n* (%)	4 (23.52)	11 (78.57)	
MV, *n* (%)	21 (95.45)	11 (47.82)	22.91 (2.88–254.80)
OMVI (mo), mean (SD)	17.33 (7.73)	*45.00 (20.93)	2.33 (0.58–8.66)
<24 mo, *n* (%)	12 (57.14)	4 (36.36)	
>24 mo, *n* (%)	9 (42.85)	7 (63.63)	
Total cholesterol at onset (mmol/L)	4.13 (0.72)	**5.31 (1.20)	7.33 (1.29–33.10)
< 4.63 mmol/L, *n* (%)	11 (73.33)	3 (27.27)	
>4.63 mmol/L, *n* (%)	4 (26.66)	8 (72.72)	

* *p* < 0.05 ** *p* < 0.01, ***p < 0.001. Differences between means of continuous variables were estimated by Student-T test or Mann-Whitney U test. OR values were calculated by bivariate logistic regression analysis. *Kg* kilograms, *mo* months, *mmol/L* millimoles per liter, *NA* not applicable, *OR* Odds ratio, *PEG* percutaneous endoscopic gastrostomy, *SD* standard deviation, 95% CI, 95% confidence interval

Other variables such as gender, presence of comorbidities, and body weight decrease after diagnosis did not affect the prognosis of ALS individuals. Nonetheless, we observed that mean survival time was longer for ALS patients younger than 60 years-old compared to older individuals, although the difference did not reach statistical significance (Table 3 and Fig. 2b).

## Discussion

Most of what it is currently known about the clinical characteristics of ALS comes from studies in white-Caucasians from Europe and North America. However, recent works have shown that epidemiology and clinical manifestations of this disease vary in relation to ethnicity of different populations [4–6]. Despite this, only few investigations have evaluated the phenotype of ALS in patients from Latin American countries [11–13]. In this context, our results provide a valuable description of the neurological characteristics and survival of Hispanic ALS patients from Mexico City. Notably, we found certain particularities in the phenotype of our population compared to what it is described in the literature. Firstly, the mean age at disease onset in the current study was lower than reported in Caucasian series from Europe [4, 14–18], Japan [19], and Israel [20], but it coincides with values observed in the United States [21], Brazil [22], and Uruguay [11]. Nonetheless, a previous investigation performed in the northern region of Mexico showed that the mean age at disease onset was around 47 years [13], much lower than observed in the current study. These discrepancies may reflect differences in the genetic background of ALS patients from distant regions around the world, and even from different places within the same country. Indeed, it is now widely recognized that some genetic abnormalities account for specific clinical phenotypes in ALS, especially among patients with the familiar form of the disease. Such genetic alterations may influence the progression of the disease as well as some clinical characteristics such as the age at symptom onset [3]. Unfortunately, our study participants were not subjected to any genetic analysis, thus we were unable to address possible genotype-phenotype relationships accounting for differences in the clinical phenotype of our population. Future studies integrating genetic data would provide an estimation of the genetic burden in Hispanic ALS patients and a better understanding of genetic factors affecting their clinical phenotype.

Secondly, we also found a higher incidence of ALS in women, and an increased percentage of bulbar ALS cases, which differs from most of previous reports [4, 6–19]. This increased proportion of bulbar onset ALS patients observed in our study could be ascribed to possible misdiagnosis of spinal onset cases, which are frequently confounded with other syndromes affecting the spinal cord and the peripheric nervous system, such as lumbar stenosis, polyneuropathy, spinal cord tumours, among other [23]. Meanwhile, the proportion of familial ALS in our population was about 5%, as described in the global literature [4].

We also addressed the effect of different clinical factors on the prognosis of ALS patients. Of note, the mean survival time observed in our population is one the longest reported in the literature [4], together with survival rates showed in another study carried out in a different region of our country [13]. As any of our study participants received treatment with riluzole, the survival rates showed here were not influenced by any disease-modifying therapy. Hence, it is possible that the differences in survival rates observed between our patients and other populations may be explained at least in part by variations in the presentation of certain predictive clinical characteristics, such as the age at disease onset and the anatomical region of symptom initiation. In this regard, we found that participants of the current study had a younger age at disease onset, which is a predictor of better survival in ALS [4]. In fact, our results showed that age at onset was inversely correlated with survival time in our population. Nonetheless, as we mentioned before, we also observed a higher proportion of ALS patients presenting with the bulbar onset form of the disease, which has been related to poor survival in previous studies as well as in the current report [4].

On the other hand, genetic factors influencing the progression of the neurological decline in ALS patients, together with environmental exposures may account for discrepancies in survival among different ethnic groups, as mentioned before. Furthermore, survival can also be influenced by differences in the nutritional support, respiratory management and other medical interventions provided to ALS patients in distinct regions. For instance, variations in the management of respiratory failure in ALS are observed between centres located in different continents [24]. Such management differences may also exist between distinct types of hospitals located in the same region. In this context, the survival rates observed in the current study may not represent the actual life expectancy of ALS patients from different regions of our country, as our data were obtained from a single third level medical centre where there might be a better access for ALS patients to get specialized management for their disease complications compared to other hospital around Mexico. Regarding other clinical predictors of survival in ALS, our results confirmed that the need for PEG and MV during the course of the disease is a predictor of poor outcome in our patients, whereas gender did not affect the overall survival of our ALS patients, contrary to other studies showing a slightly higher mortality among women [19, 22, 25]. Similarly, body weight reduction after diagnosis, a well-recognized independent predictor of mortality in ALS [26], had non predictive significance in our study.

As it has been reported in previous studies [13, 21, 25, 27], our data demonstrated that a longer diagnosis delay after onset of symptoms is a robust protective factor for mortality in ALS cases. Interestingly, we also found that patients with shorter intervals between symptom onset and diagnosis were older than individuals with longer diagnostic delay, which may explain our observations, as it is well known that there is a significantly better survival in younger ALS patients [18, 21, 25, 27]. This means that ALS patients that begin with symptoms at older ages seek medical attention earlier, but as they may have a lower capacity to compensate for the declining in motor functioning and a higher burden of other comorbidities, their life expectancy is shorter and then, their shorter interval between disease onset and diagnosis predicts a poor survival. This phenomenon may also be related to variations in the rate of disease progression among patients looking for medical attention early after disease onset compared to those attending late to their first clinical examination. In fact, Czaplinksi and colleagues have shown that the more rapidly a patient initially deteriorates the shorter the diagnostic delay [21], suggesting that patients with a faster progression of disease seek medical care earlier, whereas those with slower disease progression attend to their first examination later as they can compensate better for their initial symptoms. Apart from this, the time of diagnosis delay reported here doubles the value observed in most of the studies from Europe and the United States [4], which may reflect limitations in the diagnostic approach to ALS patients in our settings.

Finally, some studies have previously reported that the concentrations of lipids in the blood of patients with ALS measured at the time of diagnosis have a positive impact on their survival [9, 10]. Specifically, Dorst and colleagues showed that high fasting serum levels of cholesterol at baseline predicted a longer survival in a group of ALS patients [10]. Similarly, Dupuis and colleagues demonstrated that steatosis of the liver was more pronounced in ALS patients compared to individuals with Parkinson disease, and that elevated cholesterol levels increased the survival of ALS patients by more than 1 year [9]. Conversely, other studies have shown that the lipid profile has no prognostic value in ALS [28, 29], although lower serum lipids have been found to be related to respiratory impairment in ALS patients [28]. In this context, our data suggest that higher serum levels of total cholesterol at diagnosis are associated with longer survival, and lower levels correlate with increased rate of MV and PEG. Collectively, these data suggest that a greater energy reserve in the form of lipids may delay the process of motor neuron degeneration in ALS patients, who have shown an elevated resting energy expenditure [30].

Our study possesses all the restrictions of a retrospective design in relation to the access to clinical information. For instance, we could not retrieve additional data about the time of dysphagia onset in all the participants to address the impact of such variable on their survival, thus we only reported the time at which PEG was required. Similarly, forced vital capacity (FVC) was not regularly monitored in all the participants, thus we could not evaluate the predictive value of FVC at baseline nor the impact of any decrease in FVC on survival in our population [31]. Furthermore, although the primary outcome in our study was survival, we were unable to include other measurements of disease severity such as the ALS functional rating scale revised (ALSFRS-R) score to address clinical characteristics predicting functional decline in our patients [32]. Similarly, the fact that the current study was conducted at a single third-level medical centre made us unable to perform a population-based comparison, which limited our ability to estimate the local incidence of ALS and may restrict the representativeness of our data. Moreover, a better description of the ethnic background of the participants would have provided useful information for future investigations aimed to compare clinical factors and prognosis of ALS patients across populations with different genetic ancestry. Finally, a major limitation of our study is that none of the participants was subjected to genetic studies to identify some of the genetic abnormalities known to be associated with familiar and sporadic ALS as acknowledged before [3]. In this regard, although clear genotype–phenotype relationships where the presence of a specific mutation accurately predicts clinical characteristics do not exist, a better genetic characterization of our population would have provided relevant information to identify possible mutations accounting for differences in certain characteristics of the phenotype of our patients, for instance, their longer survival and their earlier age at disease onset. Despite these limitations, our study represents one of the few clinical descriptions of Hispanic ALS patients available in the literature, providing evidence of a specific phenotype in our population.

## Conclusions

In summary, the clinical characteristics and survival of Mexican ALS patients differ from the disease phenotype observed in individuals from other regions of the world. Nonetheless, the predictive value of certain clinical factors described in studies from Europe and the United States remains consistent in our population. Hence, our study may provide data of great utility to improve our understanding of survival factors in ALS among different ethnic groups, which may contribute to anticipate and individualize the diagnostic approach and therapeutic interventions in ALS patients from Mexico and other Latin American countries.

## Supplementary information

**Additional file 1:** **Figure S1.** Correlation between age at onset and ODI in ALS patients. Value of *p* and r were estimated using the Spearman correlation coefficient. mo, months; ODI, onset to diagnosis interval.

## Data Availability

The datasets used and/or analysed during the current study are available from the corresponding author on reasonable request.

## Abbreviations

95% CI: 95% confidential intervals
ALS: Amyotrophic lateral sclerosis
DDI: Diagnosis to death interval
GDI: Gastrostomy to death interval
ISSSTE: Instituto de Seguridad y Servicios Sociales de los Trabajadores del Estado
MV: Mechanical ventilation
MVDI: Mechanical ventilation to death interval
ODI: Onset to diagnosis interval
OGI: Onset to gastrotomy interval
OMVI: Onset to mechanical ventilation interval
PEG: Percutaneous endoscopic gastrostomy
SAH: Systemic arterial hypertension
SD: Standard deviation
