# Supplementary figures and images for: A retrospective study of the clinical phenotype and predictors of survival in non-Caucasian Hispanic patients with amyotrophic lateral sclerosis
Source: BMC Neurol. 2019 Oct 29;19:261. doi: 10.1186/s12883-019-1459-3 (PMC6819359; doi:10.1186/s12883-019-1459-3)

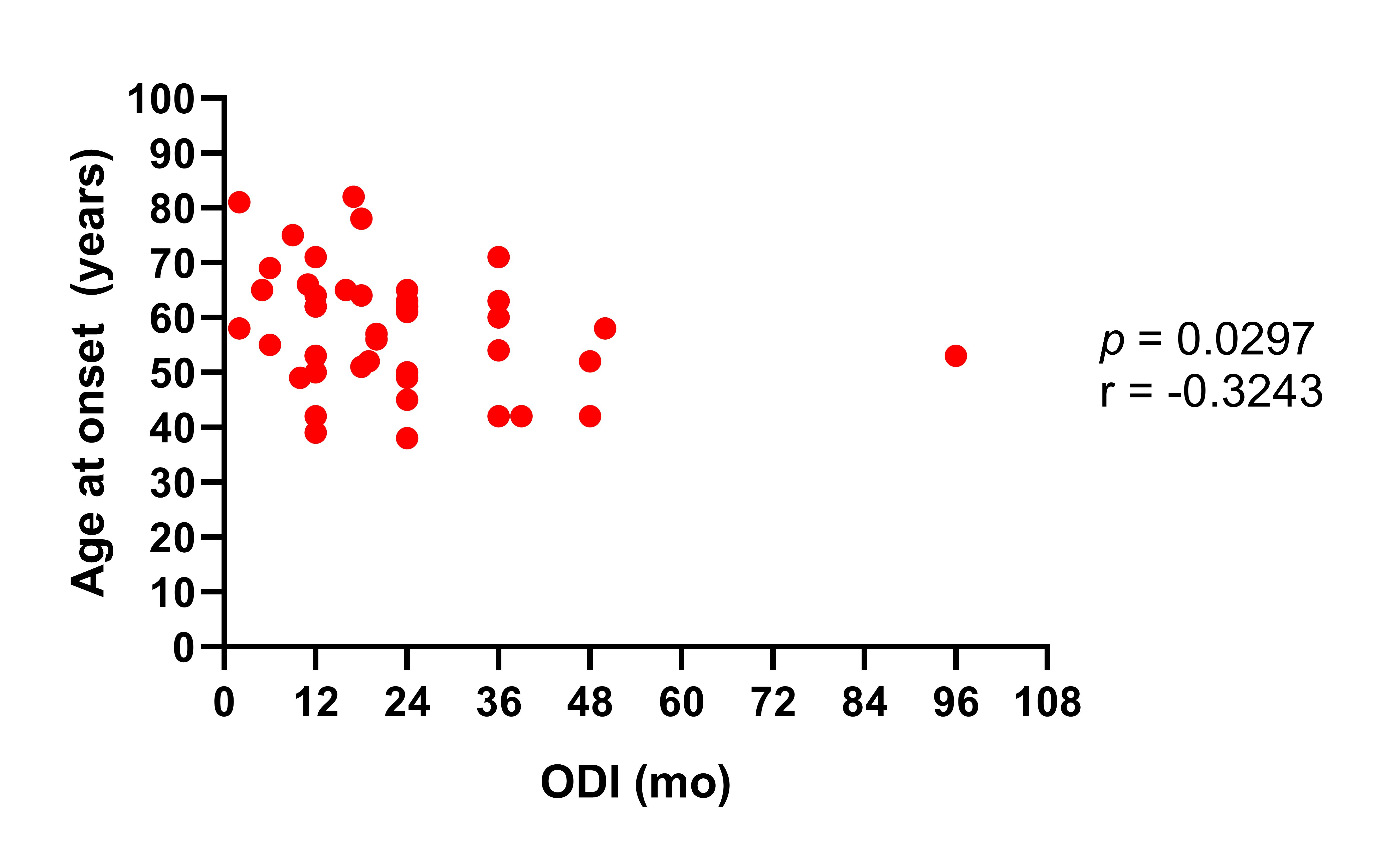

Supplement: Supplementary file 1 — Additional file 1: Figure S1. Correlation between age at onset and ODI in ALS patients. Value of p and r were estimated using the Spearman correlation coefficient. mo, months; ODI, onset to diagnosis interval. [file 12883_2019_1459_MOESM1_ESM.jpg]
